# Measured body mass index, body weight perception, dissatisfaction and control practices in urban, low-income African American adolescents

**DOI:** 10.1186/1471-2458-9-183

**Published:** 2009-06-12

**Authors:** Youfa Wang, Huifang Liang, Xiaoli Chen

**Affiliations:** 1 Center for Human Nutrition, Bloomberg School of Public Health, Johns Hopkins University, Baltimore, MD, USA; 2 Department of Human Nutrition, University of Illinois at Chicago, Chicago, IL, USA

## Abstract

**Background:**

Current understanding of the associations between actual body weight status, weight perception, body dissatisfaction, and weight control practices among low-income urban African American adolescents is limited. The knowledge can help direct future intervention efforts.

**Methods:**

Cross-sectional data including measured weight and height and self-reported weight status collected from 448 adolescents in four Chicago Public Schools were used.

**Results:**

The prevalence of overweight and obesity (BMI ≥ 85^th ^percentile) was 39.8%, but only 27.2% considered themselves as obese, although 43.4% reported trying to lose weight. Girls were more likely to express weight dissatisfaction than boys, especially those with BMI ≥ 95^th ^percentile (62.9% vs. 25.9%). BMI ≥ 85^th ^percentile girls were more likely to try to lose weight than boys (84.6% vs. 66.7%). Among all adolescents, 27.2% underestimated and 67.2% correctly judged their own weight status. Multinomial logistic models show that those with BMI ≥ 85^th ^percentile, self-perceived as obese, or expressed body dissatisfaction were more likely to try to lose weight; adjusted odds ratios and 95% confidence intervals were 4.52 (2.53–8.08), 18.04 (7.19–45.30), 4.12 (1.64–10.37), respectively. No significant differences were found in diet and physical activity between those trying to lose weight and those not trying, but boys who reported trying to lose weight still spent more television time (P < 0.05).

**Conclusion:**

Gender differences in weight perception, body dissatisfaction, and weight control practices exist among African American adolescents. One-third did not appropriately classify their weight status. Weight perception and body dissatisfaction are correlates of weight control practices. Adolescents attempting to lose weight need be empowered to make adequate desirable behavioral changes.

## Background

Nationally representative survey data show that the prevalence of overweight is about 10% higher in African American adolescent girls than in non-Hispanic white peers but is not higher in African American adolescent boys than in their white counterparts [1]. Adolescent girls and boys may evaluate their bodies differently, e.g., girls tend to view their body primarily as a means of attracting others, while boys perceive theirs as a means of effectively operating in the external environment [2]. Body weight perception is a strong determinant of nutritional habits and weight management practices among adolescents [3,4]. Body dissatisfaction is common in many adolescents in the United States, especially in adolescent girls [5]. This concern may have a number of adverse effects on their physical and psychosocial development and well-being [6-10]. Some researchers have argued that some level of body dissatisfaction may be beneficial for individuals with average or above-average weight, as it may lead to healthy weight management behaviors such as increased intake of fruits and vegetables and regular physical activity [11,12].

Several recent studies in the United States, such as the Pathways Study[13] and Project EAT [5], found that adolescents' body image was associated with their weight control intentions in certain ethnic groups. Other researches suggest the complex relationships among actual body weight, body weight perception, weight dissatisfaction, weight control intention, and actual behaviors in young people; some studies have provided conflicting evidences [3,14-16]. A common weakness of most previous studies is that the subjects' diet and physical activity are usually assessed based on their answers to a limited number of short questions. Very few studies have compared weight perception, body dissatisfaction and weight control practices in low-income minority groups and included both adolescent boys and girls.

**Figure 1:**
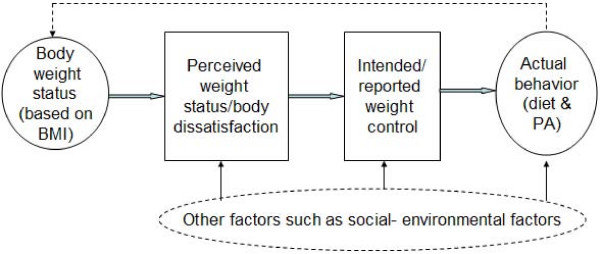
Conceptual framework: Body weight, perceived weight status, body dissatisfaction, and weight control practices*. *NOTE: The present study is not aimed to examine the impact of the "other factors" due to its scope and our available data.

The present study aimed to: 1) assess the associations between actual body weight status, body weight perception and body dissatisfaction, and weight control intention, in low-income African American adolescents; 2) study the correlates of body weight control practices; and 3) compare the differences in eating and physical activity patterns by reported weight control practices. Gender differences were examined in all aspects. Figure 1 shows our conceptual framework. We suspect that actual overweight status leads to self-perceived overweight, which in turn results in intended or reported weight control. Weight control practices, or changes in diet and physical activity, will promote weight reduction. Findings from this research will enhance our understanding of the complex relationships among these key study variables, and will provide insights in guiding the development of effective obesity prevention and control programs in adolescents, in particular, in underserved minority population groups.

## Methods

### Overview of study design

In 2003, we commenced a randomized intervention trial to test the feasibility and effectiveness of a school-based, environmental obesity prevention program in low-income African American (AA) students. Four Chicago public schools were randomly assigned to the intervention. The intervention focused on the school's environment and aimed at promoting healthy eating and physical activity (PA). The study was named the HEALTH-KIDS ("**H**ealthy **E**ating and **A**ctive **L**ifestyles from school **T**o **H**ome for **KIDS**") Study. Our school selection criteria included being located within the city of Chicago or nearby suburbs, with > 80% of students being AA, > 70% of the students from low-income families, with grades 5–8 in the same building, and with a student body that was relatively stable (mobility rate < 30%). All students in grades 5 through 7 were recruited at baseline (Fall 2003) to participate in the trial. In 2003, data were collected in the spring pilot survey and in the fall full baseline survey. The present analysis focused on the pooled spring and fall baseline data from 448 students who had complete data on anthropometric measures, body image, and other related study variables (see below), among them, 24 students' spring data were used when their fall data were not available. More details about the study design and data collection are provided elsewhere [17,18]. The study was carried out following study protocols approved by the Institutional Review Boards at the University of Illinois at Chicago and Johns Hopkins University Bloomberg School of Public Health. Informed consent was obtained from each participant.

### Data collection, measures, and study variables

Students' anthropometric measures were assessed through direct measurements conducted by trained research staff following standardized protocols in the schools. Other key data were collected in small-group surveys using self-administrated questionnaires and students were assisted by interviewers when needed. Most of the questions on weight perception, body dissatisfaction, and weight control practices were adapted from questionnaires that have been used in previous studies such as the national Youth Risk Behavior Surveillance System (YRBSS) [19], Girls Health Enrichment Multi-Site Studies (GEMS) [20], the Pathways Study [13] and the Child and Adolescent Trial for Cardiovascular Health (CATCH) [21].

#### Physical examination

Students' height was measured to the nearest 0.1 cm using a portable stadiometer (Shorr Board Stadiometer, Olney, MD), and weight was assessed to the nearest 0.1 kg for each participant in light clothing without shoes using the Tanita BWB-800 A electronic scale (Tanita Corporation, Japan). Each measure was assessed twice for each participant and the means were used in our analysis.

#### Classification of overweight and obesity

Body mass index (BMI = weight (kg)/height (m^2^)) was calculated based on measured weight and height. We used the 2000 CDC Growth Charts to define: a) overweight, 85^th ^percentile ≤ BMI < 95^th ^percentile; b) obesity, BMI ≥ 95^th ^percentile; and c) "underweight," BMI < 5^th ^percentile [22]. Due to the relatively small sample size, in some analysis we combined all those with BMI < 85^th ^percentile and called them the "non-overweight group."

#### Body weight perception and dissatisfaction

This was assessed mainly through two questions in the baseline survey: a) "How do you describe your body weight?" (answer choices: underweight, normal weight, a little overweight, very overweight); and b) "I feel bad about myself because of my weight" (answer choices: very true, a little true, not true, can't say).

#### Self-reported weight control attempt and practices

Weight control behavior was assessed with the question, "What are you trying to do about your body weight?" Provided response choices were gain weight, lose weight, stay the same, and nothing.

#### Dietary intake

Students' eating patterns were assessed by asking a number of dietary intake questions adapted from the YRBSS [19] and the CATCH [21] study questionnaires in our Health and Nutrition Questionnaire (HNQ). For example, participants were asked how often over the past seven days they had eaten fruit, vegetables, green salad, fried foods, or soft drinks as well as questions about their snack food eating patterns. In addition, their habitual eating behaviors over the past year were assessed using a 152-item food frequency questionnaire, the Harvard Youth and Adolescent Questionnaire (YAQ) [23,24]. The present study focused on the dietary questions in HNQ, because our ongoing separate analysis of the YAQ data suggested an over-reporting problem among some students (Wang and Li, 2006, unpublished). The YAQ data were only used to compare the differences in energy and nutrient intakes (could not be assessed by HNQ) between those who reported currently trying to lose weight and those who did not.

#### PA and sedentary behaviors (also called inactivity)

Students' PA and inactivity were assessed using a questionnaire adapted from the Girls Health Enrichment Multi-Site Studies (GEMS) [20] with additional questions from the YRBSS [19] questionnaire asking about children's overall PA and inactivity patterns. Changes were made to fit the needs of our study. Some examples of the overall PA questions include, "On how many of the past 7 days did you do at least 20 minutes of exercise hard enough to make you sweat and breathe hard?", "On how many of the past 7 days, did you do at least 30 minutes of light exercise that was NOT hard enough to make you sweat or breathe hard?", "On an average school day, how many hours do you watch TV or videos, or play computer or video games?" Two additional questions were asked about the students' participation in physical education (PE) class, the frequency, and how much time they actually spent exercising each time. In the present study we focused on these overall PA questions.

In order to group the participants into different PA level groups, we generated a "PA-Metabolic Equivalent (MET) Score" for each participant by combining the information collected using the two PA questions regarding hard and light exercise. MET is a unit to measure intensity of a given PA using its estimated oxygen consumption per unit time [25]. We assigned an average MET value of 6 for vigorous (or called "hard") exercise and 3 for light exercise, and then calculated the summary MET score as follows: a) vigorous exercise-score = 6*frequency*20 minutes; b) light exercise-score = 3*frequency*30 minutes; and c) PA-score = hard exercise-score + light exercise-score. The sex- and age-specific PA-score median was used to group the participants into the high-PA and low-PA groups.

### Statistical analysis

Differences in continuous variables were tested using t-tests and linear regression analysis and in categorical data using χ^2 ^tests and logistic regression models. Weighted kappa with 95% confidence intervals (CI) were calculated to measure agreement between actual and perceived body weight status. A kappa of 0.41–0.60 indicates "moderate" agreement and, 0.21–0.40, for fair agreement [26].

Multinomial logistic regression was used to examine the relationship between body weight control practices (the outcome variable) and actual body weight status, perceived body weight status, and body dissatisfaction. The referent group consisted of subjects who reported "stay the same (weight)." Analysis was stratified by measured BMI status and gender. Some researchers have argued that adjustment should be made (i.e. using smaller P values) when making multiple comparisons [27], but we chose not to do this because others disagree as to its importance and its use [28-30]. Data management and data analysis were performed with SAS Version 9.1 (SAS, Cary, NC, USA). P value was set at < 0.05 for statistically significant between-group differences.

## Results

### Patterns of body weight perception and body dissatisfaction and weight control practices

The participants' mean age was 11.9 years (SD = 1.0); 56.2% were girls. Based on measured BMI, approximately two-fifths (39.8%) were overweight or obese (39.5% in boys vs. 40.0% in girls) and 21.8% were obese (17.7% in boys vs. 25.1% in girls). However, overall only 27.2% considered themselves overweight. Much fewer boys did so than girls (19.1% vs. 33.5%). Table 1 presents the patterns of body weight perception, body dissatisfaction, and reported weight control practices by the participants' actual body weight status. There were considerable gender differences.

**Table 1:** Reported body weight perception and weight control practices among urban, low-income African American students, by measured BMI and gender

	All	Boys	Girls	Gender difference, P value*
**All participants**	(n = 448)	(n = 196)	(n = 252)	
*Self-evaluation of body weight*				
Underweight	12.3	15.4	9.8	0.002
Normal weight	60.5	65.5	56.7	
Overweight	27.2	19.1	33.5	
*Feel bad about one's body weight*				
True	24.2	15.9	30.6	0.001
Not true	66.4	73.9	60.7	
Cannot say	9.4	10.2	8.7	
*What are trying to do about your body weight?*				
Gain weight	16.3	19.1	14.1	0.524
Lose weight	43.4	40.7	45.5	
Stay the same	30.3	30.4	30.2	
Nothing	10.0	9.8	10.2	
**Participants with BMI < 85^th ^percentile**	(n = 172)	(n = 84)	(n = 88)	
*Self-evaluation of body weight*				
Underweight	19.0	22.9	16.0	0.350
Normal weight	73.1	70.3	75.3	
Overweight	7.9	6.8	8.7	
*Feel bad about one's body weight*				
True	14.7	12.1	16.9	0.337
Not true	73.8	76.8	71.3	
Cannot say	11.5	11.1	11.8	
*What are trying to do about your body weight?*				
Gain weight	25.0	27.4	23.2	0.470
Lose weight	20.9	23.9	18.5	
Stay the same	39.2	35.9	41.7	
Nothing	14.9	12.8	16.6	

	All	Boys	Girls	Gender difference, P value*

**Participants with BMI ≥ 85**^th^**percentile**				
*Self-evaluation of body weight*	(n = 178)	(n = 77)	(n = 101)	
Underweight	1.7	2.7	1.0	< 0.001
Normal weight	41.9	58.7	29.8	
Overweight	56.4	38.6	69.2	
*Feel bad about one's body weight*				
True	37.7	19.7	50.5	< 0.001
Not true	56.0	71.2	45.2	
Cannot say	6.3	9.1	4.3	
*What are trying to do about your body weight?*				
Gain weight	2.8	5.3	1.0	0.014
Lose weight	77.1	66.7	84.6	
Stay the same	17.3	22.7	13.4	
Nothing	2.8	5.3	1.0	
**Participants with BMI ≥ 95^th ^percentile** (n = 35)	(n = 98)		(n = 63)	
*Self-evaluation of body weight*				
Underweight	1.0	0.0	1.5	0.270
Normal weight	22.7	32.3	18.2	
Overweight	76.3	67.7	80.3	
*Feel bad about one's body weight*				
True	51.7	25.9	62.9	0.009
Not true	42.7	63.0	33.9	
Cannot say	5.6	11.1	3.2	
*What are you trying to do about your body weight?*				
Gain weight	0.0	0.0	0.0	0.297
Lose weight	90.7	87.1	92.4	
Stay the same	8.3	9.7	7.6	
Nothing	1.0	3.2	0.0	

*Based on chi-square tests.

### Weight perception

A large proportion of these adolescents did not have a correct perception of their weight status. Among students with BMI ≥ 85^th ^percentile, 56.4% thought that they were overweight or obese; of the overweight boys, 58.7% considered their body weight normal and 2.7% thought they were underweight, while overweight girls had a more accurate judgment: 69.2% considered themselves overweight or obese. Among students with BMI ≥ 95^th ^percentile, 23.7% did not consider themselves to be overweight or obese.

### Body dissatisfaction

One quarter of the participants (24.2%) reported body weight dissatisfaction (i.e., feeling bad about his/her body weight). Girls were twice as likely as boys to be dissatisfied (30.6% vs. 15.9%). Only 37.7% of adolescents with BMI ≥ 85^th ^percentile and half of obese (BMI ≥ 95^th ^percentile) adolescents (51.7%) expressed dissatisfaction. Overweight girls were more likely to be dissatisfied than boys.

### Weight control intention and practice

Among all adolescents, 27.2% considered themselves as overweight, but 43.4% reported trying to lose weight. Among those with BMI ≥ 85^th ^percentile, 77.1% reported trying to lose weight, higher in girls than in boys (84.6% vs. 66.7%). On the other hand, among those with BMI < 85^th ^percentile, 20.9% reported trying to lose weight.

### Agreement between actual and perceived body weight status: Did these adolescents have a good judgment of their own weight status?

Table 2 shows that overall two thirds (59.9% of boys vs. 72.8% of girls) correctly judged their own weight status. The weighted kappa statistics indicate only a moderate agreement (Kappa = 0.44); the agreement was poor in boys (Kappa = 0.32), and were significantly lower than that in girls (Kappa = 0.53, P < 0.05).

**Table 2:** Agreement between body weight perception and actual body weight status based on measured BMI among urban, low-income African American students, by gender

		Based on measured BMI*				
				
	Self-judged status	Underweight	Normal weight	Overweight or obese	Overall Agreement (%)	Weighted Kappa (95%CI)
**All**					67.2	0.44 (0.37, 0.51)
	Underweight	1.8	9.8	0.7		
	Normal weight	0.9	42.9	16.7		
	Overweight or obese	0.0	4.7	22.5		
**Boys**					59.9	0.32 (0.22, 0.42)
	Underweight	2.6	11.8	1.0		
	Normal weight	0.5	42.3	22.7		
	Overweight or obese	0.0	4.1	15.0		
**Girls**					72.8	0.53 (0.44, 0.62)
	Underweight	1.2	8.3	0.4		
	Normal weight	1.2	43.3	12.2		
	Overweight or obese	0.0	5.1	28.3		

*The figures presented in the cells are the percentages and the total for each group is 100%.

We also examined the sensitivity and specificity of self-judgment of overweight status (if used as a screening tool) against actual BMI status. The sensitivity was very low (56.4%), although the specificity was high (92.2%). The sensitivity was remarkably lower in boys than in girls (38.7% vs. 69.2%, P < 0.05), but the specificity was similar (93.3% vs. 91.3%, P > 0.05). Over a quarter (27.2%) of participants underestimated their body weight status, with more boys than girls doing so (35.6% vs. 20.9%, P < 0.001). Only 5.6% overestimated their body weight status, and no significant gender difference was found.

### Correlates of body weight control practices: Who were more likely reporting trying to lose or gain weight?

Our multinomial logistic models show that actual body weight, weight perception and dissatisfaction were significant correlates of reported body weight control practices (Table 3). Those with BMI ≥ 85^th ^percentile, perceiving being overweight, or expressing body dissatisfaction were more likely to try to lose weight; and the odds ratio (OR) and 95% CI adjusted for gender and grades were 4.52 (2.53–8.08), 18.04 (7.19–45.30), and 4.12 (1.64–10.37), respectively. Those who perceived themselves as underweight and expressed body dissatisfaction were more likely to try to gain weight (OR = 12.36 (5.57–27.44), 5.86 (2.09–16.43), respectively). The wide CIs were caused by the large variation due to small sample sizes used in OR calculation.

**Table 3:** Multinomial logistic regression models: correlates of reported body weight control practices among urban, low-income African American students*

**Correlates**	All†	Boys‡	Girls‡
			
	OR (95% CI)	OR (95% CI)	OR (95% CI)
**Body weight status and body weight perception**			
**Y = Try to lose weight**	**Model 1**	**Model 2**	**Model 3**
BMI ≥ 85^th^	4.52 (2.53, 8.08)	3.17 (1.45, 6.89)	7.22 (2.93, 17.81)
Perceived overweight	18.04 (7.19, 45.30)	5.88 (1.99, 17.33)	95.0 (11.91, 757.9)
Perceived underweight	1.58 (0.53, 4.70)	0.88 (0.21, 3.78)	3.30 (0.62, 17.57)
Feel bad about weight	4.12 (1.64, 10.37)	4.85 (1.22, 19.24)	2.98 (0.72, 12.34)
**Y = Try to gain weight**			
BMI ≥ 85^th^	0.40 (0.13, 1.22)	0.50 (0.13, 1.87)	0.21 (0.02, 2.02)
Perceived overweight	0.0002 (0.0001, 0.0003)	0.0002 (0.0001, 0.0004)	0.002 (0.001, 0.005)
Perceived underweight	12.36 (5.57, 27.44)	8.99 (3.11, 25.96)	18.86 (5.42, 65.67)
Feel bad about weight	5.86 (2.09, 16.43)	7.01 (1.42, 34.47)	4.36 (1.09, 17.48)
			
**Body weight status and misclassification**			
**Y = Try to lose weight**	**Model 4**	**Model 5**	**Model 6**
BMI ≥ 85^th^	26.33 (12.36, 56.08)	9.18 (3.64, 23.15)	112.7 (24.41, 519.9)
Overestimate weight status	14.47 (3.88, 53.92)	12.02 (1.32, 109.1)	17.62 (3.35, 92.77)
Underestimate weight status	0.21 (0.10, 0.47)	0.46 (0.18, 1.20)	0.06 (0.01, 0.30)
Feel bad about one's weight	4.81 (1.99, 11.60)	4.39 (1.10, 17.55)	4.03 (1.14, 14.22)
**Y = Try to gain weight**			
BMI ≥ 85^th^	0.08 (0.02, 0.25)	0.12 (0.03, 0.47)	0.03 (0.003, 0.29)
Overestimate weight status	1.23 (0.18, 8.48)	-§	2.56 (0.29, 22.74)
Underestimate weight status	5.92 (2.82, 12.40)	3.94 (1.45, 10.69)	10.60 (3.33, 33.76)
Feel bad about one's weight	7.84 (2.92, 21.03)	8.93 (1.97, 40.47)	5.33 (1.37, 20.70)

*Participants whose self-reported weight control practice was 'stay the same' were treated as the reference group for the outcome. The following participants were treated as references groups in the corresponding models for independent variables: perceived weight = 'normal'; feel bad about one's weight = 'no'; BMI = ' < 85^th ^percentile'; comparison of weight status between self-evaluation and that based on measured BMI = 'correspondence'.

† Gender and grade were controlled in each model; ‡ Grade was controlled in each model.

§Could not be estimated because no boy who overestimated their body weight status reported trying to gain weight. To check model fit, we had re-fit all these models by artificially assigned a boy who overestimated their body weight status to try to gain weight. All the results were consistent except for the correlates of 'overestimate weight status.'

When the discrepancies between self-perceived and measured weight status (e.g., over- and underestimation) were included in the models instead of perceived weight status alone, we found the discrepancies were significant correlates of reported body weight control practices. Students who overestimated their weight status were more likely to try to lose weight (OR = 14.47 (3.88, 53.92)), while those who underestimated their weight status were more likely to try to gain weight (OR = 5.92 (2.82, 12.40)).

### Differences in eating and physical activity patterns by reported body weight control practice: Did those who attempted to lose weight have a healthier diet and were more active?

Few of the differences in eating and physical activity behaviors between those who reported attempting to lose weight and those who did not were significant in boys or girls (Table 4). There was no significant difference in their total energy intake, although boys and girls who tried to lose weight had a lower energy intake per unit of body weight (kcal/kg) than those who did not (P < 0.001). None of other dietary intake differences between the two groups were significant. Regarding physical activity, no difference was found in boys or girls (all P > 0.05). In contrast, boys who tried to lose weight spent more time watching TV than those who did not (P < 0.05).

**Table 4:** Differences in eating and physical activity behaviors (% or mean/SD) among urban, low-income African American students, by gender and reported body weight control practices

	**Boys**			**Girls**		
		
	Try to lose weight			Try to lose weight		
		
	Yes	No	Difference, P value	Yes	Not	Difference, P value
	
***Dietary intake***						
Energy intake (kcal)	3219	3431	0.436	3119	3431	0.187
Energy intake/height (kcal/m)	20.8	23.0	0.237	20.1	22.4	0.130
Total fat (g/1000 kcal)	33.9	33.6	0.644	35.3	35.2	0.895
% of energy from fat	30.5	30.2	0.644	31.8	31.7	0.895
% of energy from animal fat	14.8	14.2	0.253	14.9	15.3	0.354
% of energy from carbohydrate	54.6	55.8	0.223	53.9	53.8	0.880
Vit C (mg/1000 kcal)	64.0	63.1	0.846	62.9	58.7	0.270
Vegetable & Fruit (servings/day)	3.2	3.5	0.664	3.3	3.0	0.540
						
***Physical activity***						
Total MET score*	899	867	0.523	875	885	0.827
More active (MET score > median)*	40.7	59.3	0.951	47.4	52.6	0.757
Total sedentary behavior (hours)	7.8	7.4	0.632	9.3	8.9	0.636
Did at least 20 min hard exercise in ≥ 5 days over the past 7 days	34.6	47.0	0.088	32.8	30.7	0.720
Did at least 30 min light exercise for ≥ 5 days over past 7 days	26.0	21.7	0.497	20.0	27.0	0.193
Screen time (watching TV or playing video games/computer) ≥ 5 hours/day	35.1	20.9	0.029†	39.7	34.8	0.432
TV time (hours, median)	4.0	2.8	0.032†	2.8	2.8	0.744

*MET: Metabolic equivalent; the PA-MET summary score was calculated based on the 40-item physical activity questionnaire;

† T-test, p < 0.05.

## Discussion

The present study systematically examined the associations between actual body weight status, weight perception, body dissatisfaction, weight control intention and practice, and actual eating and physical activity behaviors among urban, low-income, African-American adolescents. We found few differences between adolescents who reported trying to lose weight and those who did not. Our study helps add new knowledge to the growing body of related literature. The earlier large-scale Project EAT study provided important insights that enhanced our understanding of the ethnic and gender differences in adolescents' body weight perception and satisfaction and their weight control practices. However, only a local sample from urban and suburban school districts in the St. Paul/Minneapolis area of Minnesota was included in that study. Compared to our sample, they were older (34% were in middle school 7th – 8th grades and 66% in high school 9th – 12th grades), over half were white and only 19% were AA, and the majority (82.2%) were from middle and high-income families [5].

**Figure 2:**
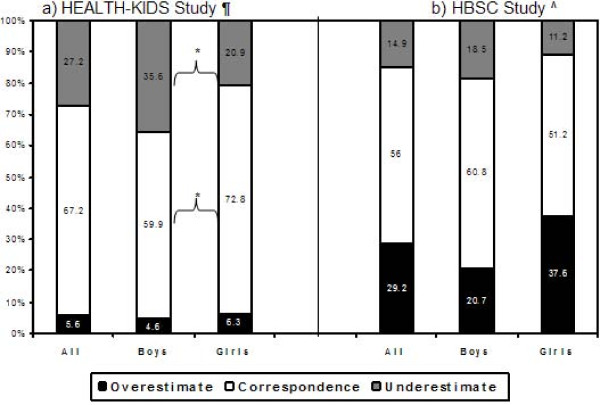
**Comparison of the discrepancies between measured and self perceived body weight status: the HEALTH-KIDS and HBSC studies**. *P < 0.05 between males and females in underestimate and correspondence. ¶ the health-kids study included 448 5–7 grade, urban low-income adolescents in chicago. ^ the HBSC study included 1826 pupils in the eighth grade of primary education and 5730 pupils in the first four years of secondary education (a total of 7556 students) in the Netherlands (ref [34]).

Among urban low-income AA adolescents in Chicago, our study found some remarkable gender differences in weight perception, body dissatisfaction, and weight control practices. First, we found that a large proportion of adolescents did not have an accurate perception of their own body weight status, especially among those with BMI ≥ 85^th ^percentile. Overall, only two-thirds (67.2%, 60% of boys and 73% of girls) correctly classified their weight status, while approximately one third underestimated their body weight. Near to half (43.6%) of those with BMI > 85^th ^percentile considered themselves as normal weight or underweight, and the figure was much higher in boys than in girls (61.4% vs. 30.8%). The discrepancies between weight perception and actual weight status clearly indicate that AA students, especially boys, underestimated their body weight. Our findings of adolescents' misclassification of their own body weight status and gender differences in body weight perception are consistent with those from previous studies [4,31,32], which have shown that body weight perceptions tend to be inaccurate when compared with BMI calculated from either self-reported or measured height and weight [4,33]. Recent studies suggest similar patterns in some other industrialized countries. For example, a study of 2,789 adolescents aged 11–14 years in the UK found that only 20% of overweight boys but 51% of overweight girls assessed their weight accurately [14]. We also compared our results with those from other countries such as a large Dutch study (the HBSC study) that included 7,556 students aged 11–16 years (Figure 2) [34]. Our students were more likely to underestimate and were much less likely to overestimate their weight status, but both studies show a gender difference. This presents a serious obstacle in the prevention and management of obesity for all adolescents, but especially for boys. The gender difference between weight perception and actual weight status supports the observation that in general Western culture gives more attention to females' weight status than to males' and also signals the need to help adolescent boys to become better informed about national weight recommendations and their own actual body weight status.

Second, a quarter (24.2%) of these students expressed body dissatisfaction, with the figure increasing among students with higher BMI percentiles and large gender differences. More than one third (37.7% in all, 19.7% of boys vs. 50.5% of girls) of those with BMI ≥ 85^th ^percentile and one half (51.7% in all, 25.9% of boys vs. 62.9% of girls) of those with BMI ≥ 95^th ^percentile expressed dissatisfaction. Other studies have also found that more girls report body weight dissatisfaction than boys [5,35,36]. For example, Project EAT reported that 46.1% of girls versus 26.1% of boys expressed low body satisfaction [5]. The remarkable gender differences are probably due to two factors: a) as noted above, more boys than girls underestimate their weight status; and b) peers and society put more pressure on girls to maintain an ideal body weight. This social pressure is also true in the target urban low-income AA communities.

Previously, a number of studies have examined the ethnic differences in body image between Caucasian and AA girls in particular [37-39], in addition to Project EAT [5]. In general, these studies show that compared to white girls, AA girls are less likely to be concerned about overweight, report that they are overweight [37], and feel pressured by society to be thin [38], though dissatisfaction with one's body weight and the desire for thinness increases as youth approach puberty [40]. Some studies have also found that AA adolescents report greater body image satisfaction and prefer to larger body sizes than do other racial and ethnic groups [36,41-44]. Project EAT shows that compared to Caucasian girls, AA girls tended to report fewer weight-related concerns, as 33.8% reported low body satisfaction compared to 46.7% of white girls, while 32.8% vs. 15.8%, respectively, expressed high body satisfaction. Girls from other minority groups (Hispanic, Asian American and Native American) tended to report similar or more concerns than Caucasian girls. Among boys, weight-related concerns and behaviors were equally or more prevalent among non-whites than among whites. This finding of significant ethnic/racial differences only in girls was also reported by others [4], and this may reflect cultural differences. In another study of 2,357 female middle and high school students, girls were asked to rate their body satisfaction regarding their height, weight, body shape, waist, hips, thighs, stomach, face, body build, and shoulders on a scale from 1 (very dissatisfied) to 5 (very satisfied). Girls who averaged 4 or higher on all 10 items were classified as "high body satisfaction." All others were designated as "not high body satisfaction." More than a quarter (27%) of adolescent girls reported high body satisfaction, with the rating most common among AA girls (40%) [45]. In the present study, as well, we found that low-income, urban, AA girls were less likely to report dissatisfaction (30.6%) compared to previous studies.

**Figure 3:**
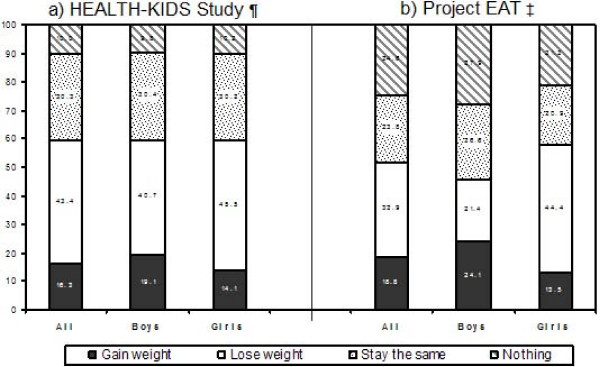
**Comparison of weight control practices in African American adolescents between the HEALTH-KIDS and the Project EAT studies**. ¶ The HEALTH-KIDS study included 448 5–7 grade, urban low-income adolescents in Chicago. ‡ Project EAT included 886 grades 7–12, urban and suburban adolescents in Minnesota (Ref [5]).

Third, we found that a large proportion (43.4%) of our study participants reported being trying to lose weight. The figure was higher in overweight and obese girls than boys (84.6% vs. 66.7%), and it was 21% even among non-overweight adolescents. These findings are consistent with those among younger girls in the National Heart, Lung, and Blood Institute (NHLBI) Growth and Health Study, i.e., approximately 40% of 2,379 black and white girls aged 9–10 years reported trying to lose weight, with the figure at 75% for those in the upper quartile of BMI [46]. However, compared to adolescents in Project EAT [5], more of our subjects were trying to lose weight and much less reported doing nothing about their weight (see Figure 3). The Project EAT included both urban and suburban AA adolescents from the 7^th ^to 12^th ^grade in Minnesota.

Our multinomial logistic regression models revealed that actual body weight status, weight perception, and body dissatisfaction were significant correlates of weight control practices. Previous studies found that lower body satisfaction predicted higher levels of dieting, unhealthy weight control behaviors, and binge eating among both boys and girls [12,33,47]. Another study reported that perception of weight was a better correlate than actual weight of whether high school students dieted or exercised to control weight [33,47]. Data collected from a nationally representative sample of adolescents aged 12 to 16 years old show that attempted weight loss was associated with perceptions of overweight independently of whether the adolescents were actually overweight [4].

Furthermore, we compared their dietary intake and physical activity level between adolescents who reported trying to lose weight and those who did not. To our knowledge, few previous investigations have examined this. Adolescents who tried to lose weight did not have higher fruit and vegetable consumption or higher physical activity compared with the others. In fact, boys who reported trying to lose weight still spent more time watching TV than those who did not. We suspect that the lack of statistical differences between them could be attributed to a few factors: those who tried to lose weight had not actually made the desirable behavioral changes; they initiated the changes but could not maintain them till the survey time; and the changes remained too small to achieve more desirable eating and physical activity patterns compared to their counterparts.

Our findings have several important implications for future primary and secondary prevention efforts. First, students and their parents need to be better informed regarding the definition of healthy body weight, and frequent assessments and professional evaluation of their growth and weight status are needed. The gender differences suggest the need for gender-tailored intervention programs. Second, adolescents who are concerned about their weight and those who have the desire to modify their weight should be provided with more appropriate and effective guidance and support to make desirable behavior changes in order to achieve their goals. The rising obesity epidemic among young people may be controlled by appropriate self-initiated weight control practices if they are supported by families, schools, health care providers, and society. As shown by our study, more than two fifths of the students were already trying to lose weight. Public health professionals should capitalize on this. Because body weight status perception is a key determinant of adolescents' weight management intention, nutrition, and physical activity habits, students who are overweight or obese but fail to perceive themselves so are unlikely to engage in appropriate weight control practices [3,4,48]. In light of the high prevalence of overweight and the many health consequences of childhood obesity, adolescents and their parents should be empowered to recognize the importance of maintaining a healthy weight and choosing healthy lifestyles following national recommendations and guidelines.

Compared to previous studies, our study has several strengths: a) We studied an underserved population group; b) Detailed information was collected regarding the participants' eating and physical activity patterns; and c) Both boys and girls were included. Previous studies in the literature on body image have been predominately conducted among girls. The present study also has several limitations. First, we studied a selective population group. Thus, the results cannot be generalized to other groups, and we cannot compare ethnic differences with our data directly. Second, causality cannot be tested due to a cross-sectional design. Third, measures of participants' behaviors were based on self-reported information, thus, measurement errors and possible reporting bias may partially explain our failure to detect more significant differences between participants trying to lose weight and the others. In addition, although diet and exercise are the most common means to control weight, other factors including genetics, family history of obesity, and stress could have influenced the results.

## Conclusion

We found considerable gender differences in body weight perception, body dissatisfaction, and their weight control practices in these urban low-income African American adolescents. A large proportion of these adolescents could not appropriately classify their weight status; and nearly 30% of them underestimated their own body weight. Body weight perception and dissatisfaction are correlates for weight control practices. Adolescents who reported trying to lose weight did not eat a healthier diet, nor were they more active compared to those who were not trying to lose weight. Overweight adolescents should be better informed and empowered to follow recommended weight loss strategies.

## Competing interests

The authors declare that they have no competing interests.

## Authors' contributions

YW designed the study, directed data collection, conducted statistical analysis and literature review, interpreted the results and wrote the manuscript. HL performed data management and statistical analysis, participated in the literature review, interpretation of the results, and writing the manuscript. XC carried out the literature review, participated in the interpretation of results and writing the manuscript. All authors read and approved the final manuscript.

## Pre-publication history

The pre-publication history for this paper can be accessed here:


